# A systematic review of outcome and outcome-measure reporting in randomised trials evaluating surgical interventions for anterior-compartment vaginal prolapse: a call to action to develop a core outcome set

**DOI:** 10.1007/s00192-018-3781-5

**Published:** 2018-10-22

**Authors:** Constantin M. Durnea, Vasilios Pergialiotis, James M. N. Duffy, Lina Bergstrom, Abdullatif Elfituri, Stergios K. Doumouchtsis, CHORUS, an International Collaboration for Harmonising Outcomes, Research and Standards in Urogynaecology and Women’s Health

**Affiliations:** 1 Department of Obstetrics and Gynaecology, Epsom and St Helier University Hospitals NHS Trust, Rowan House, Dorking Road, Epsom, London, KT18 7EG UK; 2 Nortwick Park Hospital, London North West University Healthcare NHS Trust, London, UK; 3 Laboratory of Experimental Surgery and Surgical Research N.S. Christeas, Athens University Medical School, Athens, Greece; 4 Nuffield Department of Primary Care Health Sciences, University of Oxford, Oxford, UK; 5 Balliol College, University of Oxford, Oxford, UK; 6 St George’s University of London, London, UK

**Keywords:** Anterior repair, Colporrhaphy, Core outcome sets, Cystocele, Outcomes, Outcome measures

## Abstract

**Introduction:**

We assessed outcome and outcome-measure reporting in randomised controlled trials evaluating surgical interventions for anterior-compartment vaginal prolapse and explored the relationships between outcome reporting quality with journal impact factor, year of publication, and methodological quality.

**Methods:**

We searched the bibliographical databases from inception to October 2017. Two researchers independently selected studies and assessed study characteristics, methodological quality (Jadad criteria; range 1–5), and outcome reporting quality Management of Otitis Media with Effusion in Cleft Palate (MOMENT) criteria; range 1–6], and extracted relevant data. We used a multivariate linear regression to assess associations between outcome reporting quality and other variables.

**Results:**

Eighty publications reporting data from 10,924 participants were included. Seventeen different surgical interventions were evaluated. One hundred different outcomes and 112 outcome measures were reported. Outcomes were inconsistently reported across trials; for example, 43 trials reported anatomical treatment success rates (12 outcome measures), 25 trials reported quality of life (15 outcome measures) and eight trials reported postoperative pain (seven outcome measures). Multivariate linear regression demonstrated a relationship between outcome reporting quality with methodological quality (β = 0.412; *P* = 0.018). No relationship was demonstrated between outcome reporting quality with impact factor (β = 0.078; *P* = 0.306), year of publication (β = 0.149; *P* = 0.295), study size (β = 0.008; *P* = 0.961) and commercial funding (β = −0.013; *P* = 0.918).

**Conclusions:**

Anterior-compartment vaginal prolapse trials report many different outcomes and outcome measures and often neglect to report important safety outcomes. Developing, disseminating and implementing a core outcome set will help address these issues.

## Introduction

The most common type of pelvic organ prolapse (PO) is anterior-compartment prolapse. Hendrix et al. demonstrated in a group of 16,616 postmenopausal women a prevalence of anterior-compartment prolapse of 34%, and this was much higher than the rates of apical- or posterior-compartment prolapse [1]. The aetiology of pelvic organ prolapse (POP) is complex and associated with various factors such as age, menopausal status and childbirth-related pelvic floor trauma [2, 3]. Possible surgical interventions include biological-graft, mesh and native tissue repair [4, 5]. The development of new surgical interventions is urgently required, and potential surgical interventions require robust evaluation. Selecting appropriate efficacy and safety outcomes is a crucial step in designing randomised trials. Outcomes collected and reported in randomised trials should be relevant to a broad range of stakeholders, including women with anterior-compartment prolapse, healthcare professionals and researchers. For example, resolution of bladder symptoms is an important outcome for all stakeholders; however, it is not commonly reported across trials. Even when outcomes have been consistently reported, secondary research methods, including pair-wise meta-analysis, may be limited by the use of different definitions and measurement instruments [6, 7]. A core outcome set should help address these issues. The first stage in core outcome-set development is to evaluate outcome and outcome-measure reporting across published trials. Therefore, we systematically evaluated outcome and outcome-measure reporting in published randomised trials evaluating surgical interventions for anterior-compartment prolapse. In addition, we assessed the relationships between outcome reporting quality with other important variables, including year of publication, impact factor and methodological quality.

## Materials and methods

This systematic review is part of a wider project of the International Collaboration for Harmonising Outcomes, Research and Standards in Urogynaecology and Women’s Health (CHORUS) (i-chorus.org) and was registered with the Core Outcome Measures in Effectiveness Trials (COMET) initiative database, registration number 981, and with the International Prospective Register of Systematic Reviews (PROSPERO), registration identification CRD42017062456. We searched bibliographical databases comprising the Cochrane Central Register of Controlled Trials (CENTRAL), EMBASE and MEDLINE from inception to September 2017. The search strategy used several MeSH terms, including bladder prolapse, cystocele and POP. Randomised trials evaluating surgical interventions for anterior-compartment prolapse were eligible. We included trials evaluating the surgical management of anterior prolapse as a unicompartmental prolapse procedure, as well as trials in which anterior repair was undertaken in addition to other surgical interventions. Non-randomised studies, observational studies and case reports were excluded.

Two researchers (CD and AE) independently screened the titles and abstracts of electronically retrieved articles. The articles potentially eligible for inclusion were retrieved in full text to assess eligibility, and reference lists were independently reviewed. Any discrepancies between the researchers were resolved by review of a third senior researcher (SKD). Two researchers (CD and AE) independently extracted the study characteristics, including year of publication, journal topicality (subspecialist, general obstetrics and gynaecology or general medicine), journal’s impact factor and commercial funding (yes/no). The journal’s impact factor was determined using InCites Journal Citation Reports (Clarivate Analytics, Thomson Reuters, New York, NY, USA). Funding status was identified by reviewing the article text and included the donation of equipment or other resources. Two researchers (CD and AE) independently assessed the methodological quality of included randomised trials using the modified Jadad criteria (score range 1–5) [8]. Studies were assessed as high quality when they achieved a score >4. Outcome reporting quality was assessed using the Management of Otitis Media with Effusion in Cleft Palate (MOMENT) criteria (score range 1–5) [9]. Studies were assessed as high quality when they achieved a score >4.

The non-parametric Spearman’s rank correlation coefficient (Spearman’s rho) was used to explore univariate associations between outcome reporting quality and impact factor during the year of publication, year of publication and methodological quality. Multivariate linear regression analysis using the Enter model was also undertaken to assess the combined association of quality of outcome reporting and journal type, impact factor during the year of publication, year of publication and methodological quality (independent variables) with outcome reporting (dependent variable). All tests were two-tailed. Statistical significance was set at 0.05, and analyses were conducted using the SPSS statistical software (IBM Corp. Released 2013. IBM SPSS Statistics for Windows, Version 22.0. Armonk, NY, USA).

This study was reported with reference to the Preferred Reporting Items for Systematic Reviews and Meta-Analyses (PRISMA) statement [6].

## Results

**Fig. 1:**
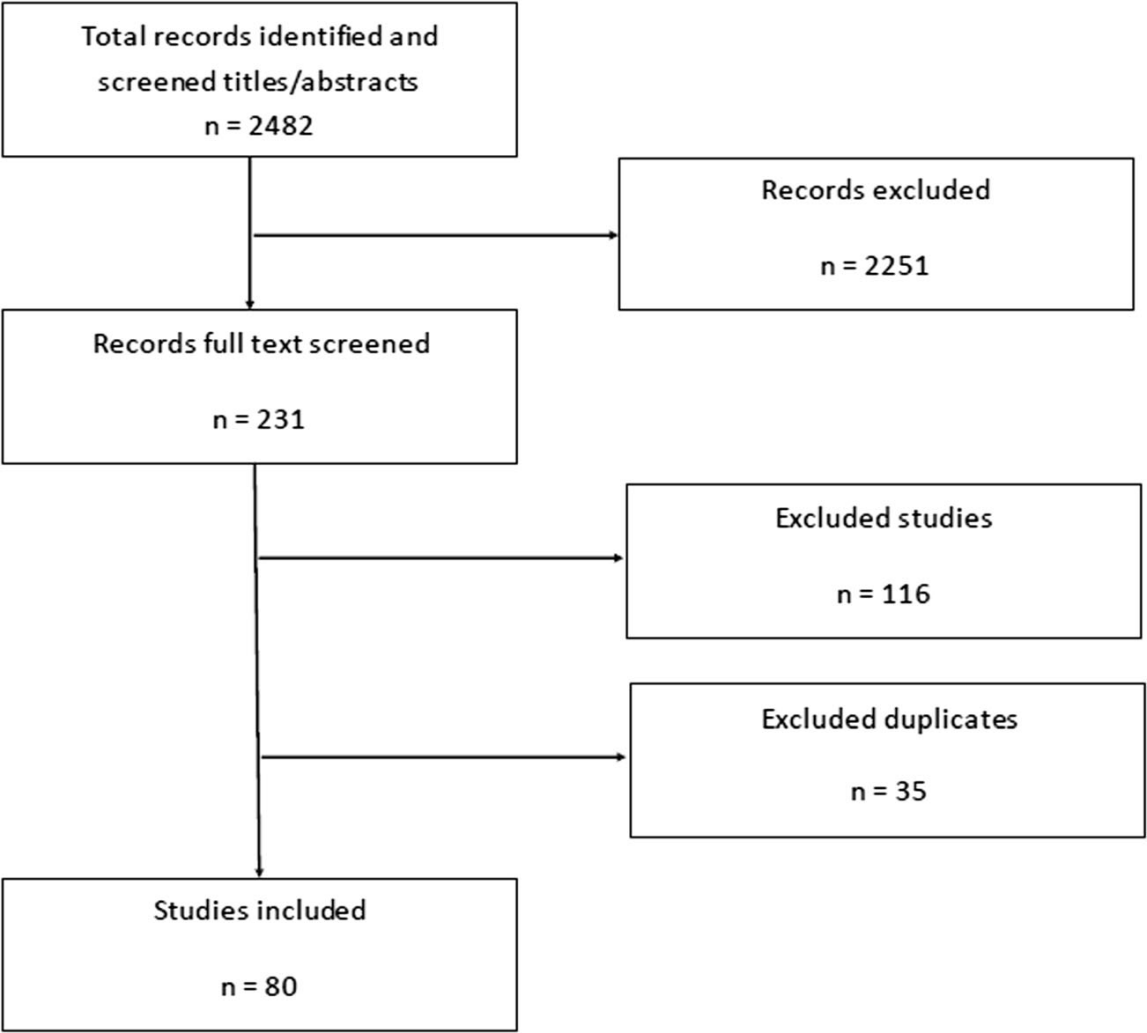
Study search and inclusion

In total, 2482 titles and abstracts were screened, and 231 potentially relevant studies were examined in detail (Fig. 1). Sixty-eight randomised trials, reporting data from 10,499 participants, met the inclusion criteria (Table 1) [5, 10–88]. Additionally, 12 randomised trials published long-term follow-up data [5, 22, 29, 39, 40, 64, 71, 72, 79, 81, 86, 87]

**Table 1:** Study characteristics

Author	Study year	Journal	Impact factor	Journal type^3^	Jadad score	MOMENT score	Study size	Commercial funding	Validated questionnaire use	Intervention group 1	Intervention group 2	Intervention group 3	Intervention group 4
Altman et al.^a^	2011	New England Journal of Medicine	29.1	G	4	5	389	Yes	Yes	Anterior colporrhaphy	Transvaginal mesh repair		
Antosh et al.	2013	Obstetrics and Gynaecology	4.78	S	3	6	60	No	Yes	Use of dilators post prolapse surgery	Non-use of dilators post prolapse surgery		
Ballard et al.	2014	International Urogynecology Journal	2.17	G	5	5	150	No	Yes	Preop. bowel preparation	Preop. non bowel preparation		
Benson et al.	1996	American Journal of Obstetrics and Gynaecology	–	S	3	3	80	No	No	Pelvic surgery for prolapse	Abdominal surgery		
Borstad et al.^a^	2009	International Urogynecology Journal	2.84	SS	3	4	184	No	No	Anterior colporrhaphy TVT	Anterior colporrhaphy + TVT staged procedure		
Bray et al.	2017	European Journal of Obstetrics & Gynaecology and Reproductive Biology	N/A	G	3	5	60	No	N/A	Suprapubic catheter	Immediate removal of catheter		
Carey et al.	2009	British Journal of Obstetrics and Gynaecology	4.64	S	3	5	139	Yes	Yes	Conventional vaginal repair	Mesh vaginal repair		
Choe et al.^a^	2000	Journal of Urology	2.64	SS	2	3	40	No	Yes	Antilogous vaginal wall slings	Micromesh		
Colombo et al.^a^	2000	British Journal of Obstetrics and Gynaecology	4.64	S	3	3	71	No	No	Anterior colporrhaphy	Burch colposuspension		
da Silveira et al.	2014	International Urogynecology Journal	2.17	SS	3	5	184	Yes	Yes	Native tissue repair	Synthetic mesh repair		
Dahlgren et al.	2011	Acta Obstetricia et Gynecologica Scandinavica	2.2	S	3	3	135	No	Yes	Conventional colporrhaphy	Porcine skin graft		
Delroy et al.^a,b^	2013	International Urogynecology Journal	2.45	SS	5	6	79	Yes	Yes	Anterior colporrhaphy	Transvaginal mesh repair		
Dias et al.^a,c^	2016	Neurourology and Urodynamics	2.48	SS	5	6	88	No	Yes	Anterior colporrhaphy	Transvaginal mesh repair		
de Tayrac et al.^a^	2012	International Urogynecology Journal	2.53	SS	3	5	147	No	Yes	Anterior colporrhaphy	Transvaginal mesh repair		
Ek et al.^a^	2012	International Urogynecology Journal	2.53	SS	2	4	99	No	Yes	Anterior trocar-guided transvaginal mesh repair	Anterior colporrhaphy with lateral defects repair		
Ek et al.^a^	2010	Neurourology and Urodynamics	3.01	SS	5	4	50	No	N/A	Anterior colporrhaphy	Trocar guided transvaginal mesh repair		
El-Nazer et al.^a^	2012	American Journal of Obstetrics and Gynaecology	1.56	S	5	5	44	No	Yes	Anterior colporrhaphy	Transvaginal mesh repair		
Farthmann et al.^a^	2013	International Urogynecology Journal	2.45	SS	3	3	200	Yes	Yes	Conventional anterior colporrhaphy	Partially absorbable mesh		
Feldner et al.^a,b^	2010	International Urogynecology Journal	2.66	SS	5	5	56	Yes	Yes	Anterior colporrhaphy	SIS graft		
Feldner et al.^a,c^	2012	Clinical Science	5.87	G	5	4	56	No	Yes	Small intestine submucosa graft	Traditional colporrhaphy		
Galvind et al.	2007	Acta Obstetricia et Gynecologica Scandinavica	1.94	G	3	2	136	No	N/A	3-h catheterisation and vaginal tampon	24-h catheterisation and vaginal tampon		
Gandhi et al.^a^	2005	American Journal of Obstetrics and Gynaecology	4	S	3	5	154	No	No	Anterior colporrhaphy	Colporrhaphy and fascial patch		
Geller et al.	2011	British Journal of Obstetrics and Gynaecology	4.34	S	3	4	50	No	N/A	Spontaneous postop. micturition	Micturition after bladder refill		
Glazener et al.^b^	2017	The Lancet	N/A	G	3	6	1352	No	Yes	Standard repair	Mesh repair	Biological graft	
Glazener et al.^c^	2017	Health Technology Assessment	N/A	G	4	6	3087	No	Yes	Standard repair	Mesh repair	Biological graft	
Guerette et al.^a^	2009	Obstetrics and Gynaecology	4.69	S	4	4	94	Yes	Yes	Anterior repair	Anterior repair + porcine graft mesh		
Gupta et al.^a^	2014	South African Journal of Obstetrics & Gynaecology	0.23	S	3	4	106	No	N/A	Anterior repair	Anterior repair + mesh		
Hakvoort	2004	British Journal of Obstetrics and Gynaecology	4.75	S	2	3	100	No	N/A	4-day catheterisation	1-day catheterisation		
Henn et al.	2016	International Urogynecology Journal	1.83	SS	5	6	80	No	N/A	Vaginal vasoconstrictor infiltration	Vaginal saline infiltration		
Hiltunen et al.^a,b^	2007	Obstetrics and Gynaecology	4.45	G	3	4	202	No	No	Anterior colporrhaphy	Transvaginal mesh repair		
Nieminen et al.^a,c^	2010	American Journal of Obstetrics and Gynaecology	4.98	G	3	4	202	No	No	Anterior colporrhaphy	Transvaginal mesh repair		
Nieminen et al.^a,c^	2008	International Urogynecology Journal	2.51	SS	3	2	202	No	No	Anterior colporrhaphy	Transvaginal mesh repair		
Huang et al.	2010	International Urogynecology Journal	2.66	SS	3	3	90	No	N/A	Removal of catheter on day 2 postop.	Removal of catheter on day 3 postop.	Removal of catheter on day 4 postop.	
Hviid et al.^a^	2010	International Urogynecology Journal	2.66	SS	3	3	61	No	Yes	Conventional anterior repair	Anterior repair + porcine skin collagen implants		
Iglesia et al.	2010	Obstetrics and Gynaecology	4.98	S	5	6	65	No	Yes	Conventional colporrhaphy or uterosacral ligament suspension	Vaginal colpopexy with mesh		
Kamilya et al.	2010	Journal of Obstetrics and Gynaecology Research	1.13	S	3	6	200	No	N/A	Catheter removal day 4 postop.	Catheter removal day 1 postop.		
Khalil et al.	2016	Journal of Clinical Anaesthesia	1.64	S	5	5	57	No	No	General anaesthesia	General anaesthesia + pudendal nerve block		
Kringel et al.^a^	2010	International Urogynecology Journal	2.66	SS	3	5	232	No	N/A	Intraurethral catheterisation 24 h	Intraurethral catheterisation 96 h	Suprapubic catheterisation 96 h	
Lambin et al.^a^	2013	International Urogynecology Journal	2.45	SS	3	5	68	No	Yes	Anterior colporrhaphy with vaginal colposuspension	Transvaginal mesh repair		
Lazzeri et al.^a^	2007	Journal of Urology	4.27	S	3	5	47	No	Yes	Abdominal prolapse repair NO Burch colposuspension	Abdominal prolapse repair and Burch colposuspension		
Lindholm et al.	1985	International Journal of Gynaecology and Obstetrics	N/A	S	4	3	20	No	N/A	Phenoxybenzamine use	Control		
Mahuvrata et al.	2011	Journal of Obstetrics and Gynaecology	0.75	G	5	5	66	No	Yes	Mesh repair	No mesh	PDS	Vicryl
McNanley et al.	2012	Female Pelvic Medicine & Reconstructive Surgery	0.42	SS	3	6	60	No	Yes	Docusate sodium laxative postoperative	Other laxatives postoperative		
Menefee et al.^a^	2011	Obstetrics and Gynaecology	5.34	S	5	6	99	Yes	Yes	Anterior colporrhaphy	Mesh repair	Biological graft	
Meschia et al.^a^	2003	American Journal of Obstetrics and Gynaecology	2.96	S	3	5	50	No	No	Endopelvic fascia plication	TVT + Anterior repair		
Minassian et al.^a^	2014	Neurourology and Urodynamics	2.71	SS	3	5	70	No	Yes	Conventional anterior colporrhaphy	Abdominal paravaginal defect repair		
Miranda et al.^a^	2011	Journal of obstetrics and gynaecology Canada	1.42	S	5	2	22	No	N/A	Anterior colporrhaphy with polyglactin 910 mesh	Anterior colporrhaphy without plication of pubovesical fascia		
Natale et al.^a^	2009	International Urogynecology Journal	2.84	SS	3	5	190	No	Yes	Anterior colporrhaphy	Synthetic mesh		
Park et al.^a^	2013	International Urogynecology Journal	2.45	SS	3	5	92	No	Yes	Anterior repair + TVT	TVT		
Pauls et al.	2015	American Journal of Obstetrics and Gynaecology	5.23	S	5	5	74	No	Yes	Dexamethasone prior to surgery	Placebo		
Ploege et al.	2015	International Urogynecology Journal	1.83	SS	3	6	91	Yes	Yes	Prolapse surgery	Prolapse surgery + TVT		
Qatawneh et al.	2013	Gynaecological Surgery	0.46	S	3	5	116	No	No	Native tissue repair	Mesh repair		
Quadri et al.^a^	2000	International Urogynecology Journal	1.15	SS	3	3	45	No	N/A	Use of PGE-2	Control		
Robert et al.^a^	2014	Obstetrics and Gynaecology	4.76	S	5	4	57	Yes	Yes	Anterior colporrhaphy	Transvaginal mesh repair		
Rudnicki et al.^a,b^	2013	British Journal of Obstetrics and Gynaecology	2.9	G	3	5	160	No	Yes	Anterior colporrhaphy	Transvaginal mesh repair		
Rudnicki et al.^a,c^	2015	British Journal of Obstetrics and Gynaecology	2.9	G	3	3	138	No	Yes	Anterior colporrhaphy	Transvaginal mesh repair		
Sand et al.	2001	American Journal of Obstetrics and Gynaecology	2.72	S	3	4	161	No	N/A	Conventional anterior colporrhaphy	Use of mesh		
Schierlitz et al.	2013	International Urogynecology Journal	2.45	SS	3	5	80	No	Yes	Conventional pelvic repair	Conventional pelvic repair + TVT		
Segal et al.	2006	International Urogynecology Journal	2.38	SS	3	5	40	No	No	Local anaesthesia	General anaesthesia		
Sivaslioglu et al.^a^	2007	International Urogynecology Journal	2.79	SS	3	2	90	No	Yes	Anterior colporrhaphy	Transvaginal mesh repair		
Stekkinger et al.	2011	Gynecologic and Obstetric investigation	1.74	G	3	5	126	No	N/A	Trans urethral catheter	S/pubic catheter		
Tamanini et al.^a,b^	2012	International Braz J Urol: official journal of the Brazilian Society of Urology	1.24	G	4	5	100	No	Yes	Anterior colporrhaphy	Transvaginal mesh repair		
Tamanini et al.^a,c^	2012	International Braz J Urol: official journal of the Brazilian Society of Urology	1.24	G	4	5	100	No	Yes	Anterior colporrhaphy	Transvaginal mesh repair		
Tamanini et al.^a,c^	2014	Journal of Urology	4.68	S	4	5	92	No	Yes	Anterior colporrhaphy	Transvaginal mesh repair		
Tantanasis et al.^a^	2008	Acta Obstetricia et Gynecologica Scandinavica	1.72	S	2	2	50	No	No	Anterior colporrhaphy	Bladder base tape repair		
Thiagamoorthy et al.	2013	International Urogynecology Journal	2.45	SS	5	6	190	No	N/A	Use of postop. vaginal pack	No use of postop. vaginal pack		
Tincello et al.^a^	2009	British Journal of Obstetrics and Gynaecology	4.18	S	3	4	31	No	Yes	Colposuspension + anterior repair	TVT + Anterior repair		
Turgal et al.^a^	2013	European Journal of Obstetrics & Gynaecology and Reproductive Biology	2.4	G	3	2	40	No	No	Anterior colporrhaphy	Transvaginal mesh repair		
Van et al.	2011	International Urogynecology Journal	2.39	SS	3	5	179	No	N/A	1-day suprapubic catheterisation	3-day suprapubic catheterisation		
Vollebregt et al.^a,b^	2011	British Journal of Obstetrics and Gynaecology	2.96	S	5	6	125	No	Yes	Anterior colporrhaphy	Transvaginal mesh repair		
Vollebregt et al.^a,c^	2012	Journal of Sexual Medicine	3.67	SS	5	6	125	No	Yes	Anterior colporrhaphy	Transvaginal anterior or posterior mesh repair		
Weber et al.^a,b^	2001	American Journal of Obstetrics and Gynaecology	2.72	G	2	3	114	No	No	Unilateral anterior colporrhaphy	Anterior colporrhaphy	Transvaginal mesh repair	
Chmielewski et al.^a,c^	2011	American Journal of Obstetrics and Gynaecology	5.34	G	4	4	114	No	No	Unilateral anterior colporrhaphy	Anterior colporrhaphy	Transvaginal mesh repair	
Weemhoff et al.^a^	2011	International Urogynecology Journal	2.39	SS	3	6	246	No	N/A	Postop. catheterisation for 2 days	Postop. catheterisation for 5 days		
Wei et al.^a^	2012	New England Journal of Medicine	29.36	G	5	6	337	No	Yes	Anterior repair	TVT + Anterior repair		
Westermann et al.	2016	Female Pelvic Medicine & Reconstructive Surgery	1.49	SS	4	5	93	No	Yes	Use of postop. vaginal pack	No use of postop. vaginal pack		
Withagen et al.^b^	2011	Obstetrics and Gynaecology	5.34	S	5	6	194	No	Yes	Conventional colporrhaphy	Transvaginal mesh repair		
Withagen et al.^c^	2011	British Journal of Obstetrics and Gynaecology	4.34	S	5	6	59	No	Yes	Conventional colporrhaphy	Transvaginal mesh repair		
Milani et al.^c^	2011	Journal of Sexual Medicine	3.67	SS	3	6	59	No	Yes	Conventional colporrhaphy	Trocar-guided Mesh		
Yuk et al.^a^	2012	Journal of Minimally Invasive Gynaecology	2.1	S	3	3	87	No	N/A	2-point mesh	4-point mesh		

*SS* subspecialty (urogynaecology),* S* specialty (obs/gyn),* G* general,* TVT* tension free vaginal tape (retropubic tape), * PDS* polydioxanone

^a^Studies focused on surgical management of anterior repair solely, ^b^original study, ^c^secondary analysis

.

Trials were published between 1985 and 2017, with most being published in subspecialty journals (33/80; 41%). Trials were frequently published in journals with an impact factor <3 [median = 2.7; interquartile range (IQR) = 2.2–4.3] and were generally small (median = 93; IQR = 60–154). Ten trials (14%) declared commercial funding. The methodological quality and outcome reporting quality varied considerably between trials (Table 1). One hundred different outcomes were organised into 11 thematic domains. The three most commonly reported thematic domains were presence of symptoms posttreatment (50 trials, 28 outcomes; 28 outcome measures), prolapse treatment success rates (47 trials; 3 outcomes; 16 outcome measures) and perioperative complications (46 trials; 15 outcomes; 13 outcome measures) (Table 2). Commonly reported outcomes were anatomical prolapse stage (43 trials; 54%), commonly assessed using the Pelvic Organ Prolapse Quantification (POP-Q) instrument (35 trials; 81%), QoL (25 trials; 31%); and intra- and postoperative complications (23 trials; 29%). Patient-reported outcomes were infrequently reported; for example, a minority of trials reported prolapse symptoms (9 trials; 11%), urinary symptoms (11 trials; 14%) and sexual dysfunction (14 trials; 17%) (Table 3). Eleven trials (14%) reported patient satisfaction.

**Table 2:** Most commonly reported outcome domains

Outcome domains	RCTs reporting on the domain	Outcomes reported	Outcome measures reported
Presence of symptoms posttreatment	50	28	28
Prolapse treatment success rate	47	3	16
Perioperative complications and observations	46	15	13
Quality of life and satisfaction with treatment	40	5	25
Treatment success evaluation	15	11	–
Postoperative catheterisation	10	17	10
Pain	9	4	7
Mesh-related outcomes	8	3	–

*RCT* randomised controlled trial

**Table 3:** Outcomes reported in 80 randomised controlled trials (RCTs) evaluating surgical management of anterior-compartment prolapse

Outcomes	Reporting studies
Prolapse treatment success rate	
Anatomical prolapse stage	43
Composite anatomical/functional success rate	3
Urethral mobility	1
Perioperative complications and observations	
Complications intra-/postoperatively	23
Postoperative hospital stay length	11
Blood loss intraoperatively	6
Duration of operation	6
Quality and time of recovery	4
Postoperative nausea and vomiting	3
Bleeding postoperatively (with/out vaginal pack use)	2
Constipation preoperatively	2
Blood pressure	2
Blood transfusion indicated	2
Heart rate change	2
Consistency of bowel movement postoperatively	1
Intra- and postoperative morbidity	1
Time to first postoperative bowel movement	1
Time to mobilisation	1
Pain	
Postoperative pain	8
Intraoperative requirement of analgesics	1
Total analgesic consumption	1
Pain level associated with first postoperative bowel movement	1
Postoperative catheterisation	
Postoperative UTI	5
Recatheterisation rates	5
Postoperative catheterisation duration	4
First postvoid residual volume	4
Time to normal spontaneous voiding	2
Acute urinary retention	1
Bacterial count in the urine	1
Catheter blockage	1
Day of spontaneous voiding	1
Diagnostic accuracy of different voiding trial methods	1
Mean residual urine volume pre- and postoperatively	1
Prediction of voiding dysfunction lasting >7 days.	1
Prolonged catheterisation	1
Pyelectasia	1
Residual urine volume	1
Urinary retention prevention with intravesically administered prostaglandin-E2	1
Urinary retention rates	1
Postoperative vaginal packing	
Bleeding postoperatively (with/out vaginal pack use) (compared with menstrual average)	1
Bleeding postoperatively (with/out vaginal pack use)	1
Presence of vaginal haematoma	1
Presence of vaginal infection	1
Bother related to the pack	1
Presence of symptoms posttreatment	
Sexual dysfunction symptoms	14
Urinary symptoms	11
Prolapse symptoms postoperatively	9
Dyspareunia	6
SUI postoperatively	5
De novo SUI postoperatively	4
Change in urinary symptoms (any)	3
Prolapse symptoms severity	3
De novo urinary urgency	2
Postoperative urinary symptoms	2
Urinary symptoms severity	2
Bowel symptoms	2
Faecal incontinence	2
Postoperative bowel symptoms	2
Change in incontinence rates	1
De novo urinary symptoms	1
De novo voiding difficulty	1
Urgency and urge urinary incontinence	1
Worsening urinary symptoms (any)	1
Obstructed defecation	1
Back pain improvement	1
Change in a pelvic symptom score	1
Change of vaginal symptoms	1
Symptomatic prolapse improvement	1
Time of prolapse recurrence	1
De novo dyspareunia	1
Sexual function in partner	1
QoL and satisfaction with treatment	
QoL and impact from symptoms evaluation	25
Patient satisfaction with treatment	11
Surgeon satisfaction with operation	2
Patient acceptability of preoperative bowel preparation	1
Surgeon—ease of procedure	1
Treatment success evaluation	
Symptoms—presence posttreatment	5
Subjective cure rates	3
Cure of SUI postoperatively	3
Reoperation rates	3
Symptoms—bother change	2
Retreatment success rates	1
Symptom improvement	1
Functional recurrence	1
Healing abnormalities	1
Need for subsequent anti-incontinence surgery	1
Treatment of overactive bladder	1
Mesh-related outcomes	
Mesh erosion	6
Mesh shrinkage	2
Degree of morbidity in mesh vs. native tissue	1
Cost/effectiveness	
Cost-effectiveness of treatment	2
Cost of procedure	1
Recruitment feasibility	
Number of patients agreed to participate	1
Number of eligible patients	1
Physician acceptance and protocol	1
Rate of recruitment compliance	1

*UTI* urinary tract infection,* SUI* stress urinary incontinence, *QoL* quality of life

Forty-two randomised trials compared native tissue or biological graft versus mesh repair for anterior vaginal prolapse. Mesh-related complications were rarely reported: seven trials (9%) reported mesh erosion, six (7%) reported mesh shrinkage and a single trial (1%) reported the degree of morbidity associated with mess Only three trials (4%) evaluated cost effectiveness. One hundred and twelve different outcome measures wer reported (Table 4). Forty-six questionnaires were used as measurement instruments, most of which were validated (45; 98%). Anterior prolapse symptoms were measured using the Pelvic Organ Prolapse Urinary Incontinence Sexual Questionnaire (PISQ-12) (13 trials; 16%), Urogenital Distress Inventory (UDI-6) (11 trials; 14%) and the Pelvic Floor Distress Inventory (PFDI-20) (9 trials; 11%). QoL was measured using the Prolapse Quality of Life (P-QoL) (10 trials; 12%), Pelvic Floor Impact Questionnaire Short Form (PFIQ-7) (8 trials; 10%) and the Incontinence Impact Questionnaire Short Form (IIQ-7) (6 trials; 7%). Table 5 summarises our main findings, demonstrating the most frequently reported outcomes. It reveals the significant discrepancies in terms of outcome reporting.

**Table 4:** Outcome measures reported in 80 randomised controlled trials (RCTs) evaluating surgical management of anterior-compartment prolapse

Outcomes	No of reporting studies
Prolapse treatment success rate	
Anatomical success rate POP-Q < 2	23
Anatomical success rate (POP-Q ≤ 1)	5
Anatomical success rate (postoperative POP-Q stage improvement)	5
Anatomical success rate (POP above hymen)	3
Anatomical success rate POP-Q ≤ 2	2
Anatomical success rate (POP-Q < 2 vs. POP-Q ≤ 1)	1
Anatomical success rate POP-Q Index (POP-Q-I) = 0	1
Anatomical success rate (postoperative POP-Q + BW stage improvement)	1
Anatomical success rate (cotton swab mobility test)	1
Composite success rate (POP-Q < 2 + UDI question 16 negative	1
Composite success rate (POP above hymen + VAS >20 (0–100 scale))	1
Composite success rate - (POP above hymen + no symptoms)	1
Composite success rate - (apex below levator plate + no symptoms)	1
Denovo POP in untreated compartments (POP-Q ≥ 2)	1
Denovo POP in untreated compartments (POP ≥ hymen)	1
Recurrence rate of POP (halfway BW stage change)	1
Perioperative complications and observations	
Postoperative hospital stay length (days)	11
Blood loss (ml)	8
Duration of operation (min)	6
PONV (postoperative nausea and vomiting), visual analogue scale [VAS (0–10)]	2
PONV scale	2
PONV QoR (quality of recovery) score > 50	2
Recovery time (days)	2
PONV intensity score [QoR (0–40)]	1
Blood pressure (mmHg)	1
Heart rate (beats/min)	1
Consistency of bowel movement (Bristol stool scale)	1
Constipation perioperatively (Rome III constipation questionnaire)	1
Time to mobilisation (days)	1
Pain	
VAS (0–10)	5
VAS (0–100)	2
VAS (not specified)	2
Mcgill pain questionnaire	2
Verbal numerical pain scale (0–10)	1
Baudelocque’s questionnaire	1
Nonvalidated questionnaire (0–3)	1
Postoperative catheterisation	
Postoperative catheterisation duration (days)	4
Day of spontaneous voiding (days)	3
Bacterial count in the urine	1
Residual urine volume (ml)	1
First PVR (postvoid residual volume) > 150 ml	1
First PVR > 1500 ml	1
Mean residual urine volume pre- and postoperatively (ml)	1
Recatheterisation if PVR >200 ml	1
Prediction of voiding dysfunction >7 days (positive predictive value)	1
Diagnostic accuracy of two voiding trial methods (sensitivity/specificity)	1
Postoperative vaginal packing	
Bleeding postoperatively (with/out vaginal pack use) (compared with menstrual average)	1
Bleeding postoperatively (with/out vaginal pack use) [FBC change and volume (ml)]	1
Blood pressure (mmHg)	1
Heart rate (beats/min)	1
Blood transfusion indicated (yes/no)	1
Vaginal haematoma (TVUSS)	1
Vaginal infection (HVS)	1
Bother related to the pack (VAS 0–100)	1
Presence of symptoms posttreatment	
PISQ-12 (Pelvic Organ Prolapse Urinary Incontinence–Sexual Questionnaire)	13
UDI-6 (Urogenital Distress Inventory)	11
PFDI-20 (Pelvic Floor Distress Inventory)	9
SUI urodynamic studies	7
DDI (Defecatory Distress Inventory)	5
ICIQ-UI SF (International Consultation on Incontinence Questionnaire–Short Form)	4
SUI cough test (presence of leakage)	4
FSFI (Female Sexual Function Index)	2
ICIQ-BS (International Consultation on Incontinence Questionnaire–Bowel Symptoms)	2
PGI-I (Patient Global Impression of Improvement)	2
OAB-V8 (Overactive Bladder-Validated 8-question)	2
POPDI-6 (Pelvic Organ Prolapse Distress Inventory)	2
POP-SS (Pelvic Organ Prolapse Severity of Symptoms)	2
UDI-I (Urogenital Distress Inventory–Irritative)	2
UDI-O (Urogenital Distress Inventory-Obstructive)	2
UDI-S (Urogenital Distress Inventory–Stress)	2
AUASS [American Urological Association Symptom Score (urinary)]	1
CRADI-8 (Colorectal–Anal Distress Inventory)	1
CRAIQ-7 (Colorectal–Anal Impact Questionnaire)	1
Danish prolapse questionnaire	1
ICIQ-VS (International Consultation on Incontinence Questionnaire–Vaginal Symptoms)	1
MESAAQ (Medical Epidemiologic and Social Aspects of Ageing Questionnaire)	1
MHU (French Urinary Dysfunction Measurement Scale)	1
MSHQ (Male Sexual Health Questionnaire)	1
PGI-S (Patient Global Impression of Severity)	1
QS-F (Sexual Quotient–Female Version)	1
SUI number of daily pads	1
Impact on quality of life	
P-QoL (Prolapse Quality of Life)	10
PFIQ-7 (Pelvic Floor Impact Questionnaire–Short Form)	8
IIQ-7 (Incontinence Impact Questionnaire–Short Form)	6
ICIQ-UI SF (International Consultation on Incontinence Questionnaire–Urinary Symptoms)	4
ICIQ-VS (International Consultation on Incontinence Questionnaire–Vaginal Symptoms)	3
KHQ (King’s Health Questionnaire)	3
UIQ-7 (Urogenital Impact Questionnaire)	3
DDI (Defecatory Distress Inventory)	2
EQ5D [Quality of Life (EuroQol)]	2
POPIQ-7 (Pelvic Floor Impact Questionnaire–Prolapse)	2
VAS (0–10)	2
CRAIQ-7 (Colorectal–Anal Impact Questionnaire)	1
PSI-QOL (Prolapse Symptom Inventory and Quality of Life Questionnaire)	1
SF-12 (12-Item Short-Form Health Survey)	1
SF-36 (36-Item Short-Form Health Survey)	1
Satisfaction	
Patient satisfaction with treatment, VAS (0–10)	3
Patient satisfaction with treatment, PGI (Patient Global Improvement)	3
Patient satisfaction with treatment (yes/no)	3
Patient satisfaction with treatment, VAS (0–100)	2
Patient satisfaction with treatment, VAS (0–4)	1
Patient satisfaction with treatment, custom (0–5)	1
Patient acceptability of preoperative bowel preparation, VAS) (0–4)	1
Surgeon satisfaction with preoperative bowel preparation, Likert scale (0–4)	1
Surgeon ease to perform operation, Likert scale (0–4)	1
Surgeon’s satisfaction with operation, VAS (0–100)	1
Cost/effectiveness	
Incremental cost per quality-adjusted life-year (QALY)	2
Cost of procedure (US$)	1

*TVUSS* transvaginal ultrasound scan, *HVS* high vaginal swab, *FBC* full blood count

**Table 5:** Reported outcomes by by more than eight studies with greater than 93 participants (median value)

Study	Sample size (*N*)	Outcomes								
		Anatomical prolapse stage	Quality of life and impact from symptoms	Complications intra-/postoperatively	Sexual dysfunction symptoms	Postoperative hospital stay length	Urinary symptoms	Patient satisfaction with treatment	Prolapse symptoms postoperatively	Postoperative pain
Glazener et al.	1352	x		x	x		x	x	x	
Altman et al.	389	x		x	x					
Wei et al.	337		x	x						
Weemhoff et al.	246					x				
Nieminen et al.	203				x				x	
Hiltunen et al.	202	x		x						
Farthmann et al.	200	x						x		x
Kamilya et al.	200					x				
Withagen et al.	194	x	x	x		x				x
Natale et al.	190	x	x							
Thiagamoorthy et al.	190									x
da Silveira et al.	184	x			x					
Borstad et al.	184	x								
Van et al.	179					x				
Sand et al.	161	x								
Rudnicki et al.	160	x	x	x						
Gandhi et al.	154	x								
Ballard et al.	150							x		
de Tayrac et al.	147	x		x				x		
Carey et al.	139	x	x	x				x		
Rudnicki et al.	138	x								
Dahlgren et al.	135	x			x		x		x	
Stekkinger et al.	126					x				
Vollebregt et al.	125	x	x	x	x					
Qatawneh et al.	116	x		x				x	x	
Weber et al.	114	x		x						
Chmielewski et al.	114	x								
Gupta et al.	106	x		x						
Tamanini et al.	100	x	x		x				x	
Hakvoort	100					x				
Menefee et al.	99	x	x		x				x	
Ek et al.	99	x								
Guerette et al.	94	x	x		x		x			
Westermann et al.	93							x		x
Studies not included	<93	19	16	11	5	5	8	4	3	4
Total studies		43	25	23	14	11	11	11	9	8

We observed a moderate correlation between outcome reporting quality and year of publication in the univariate analysis (*r* 0.458; *p*  < .001) and study quality (*r* 0.409; *p*  < .001) (Table 6). The latter index significantly affected outcome reporting in the multivariate logistic regression (β = 0.412; *p*  = .018).

**Table 6:** Univariate and multivariate correlation with outcome reporting quality

Factor	Univariate		Multivariate	
	Spearman’s rho	*P* value	Beta	*P* value
Study quality (Jadad)	00.409	**<0.001**	0.412	**0.018**
Journal IF	0.053	0.643	0.078	0.306
Year of publication	0.458	**<0.001**	0.149	0.295
Study size	0.215	0.051	0.008	0.961
Journal type	–	–	0.024	0.852
Commercial funding	–	–	−0.013	0.918
Validated questionnaire	–	–	1.310	0.196

Bolded data statistically significant

## Discussion

### Summary of main findings

This study demonstrated considerable variation in outcome and outcome-measure reporting across published trials evaluating surgical interventions for anterior-compartment prolapse. Commonly reported outcomes included normalised anatomy, QoL and pain. Patient-reported outcomes were infrequently reported, and a minority of trials reported on patient satisfaction. Mesh-related complications, including erosion, shrinkage and morbidity, were rarely reported. Forty-five different questionnaires were used as measurement instruments; most were validated. Only a few trials considered cost effectiveness.

### Strengths and limitations

Strengths of our systematic review include originality, a rigorous search strategy and methodological robustness. To our knowledge, this systematic review is the first to evaluate outcomes and outcome measures in anterior-compartment prolapse trials. Study screening and selection and data extraction and assessment were conducted independently by two researchers to avoid bias. Our findings were based on outcome reporting in published randomised trials. The exclusion of observational studies may have potentially missed outcomes related to harm [89, 90] and selecting only trials reported in English may have introduced selection bias. The variation of interventions for correcting anterior prolapse may have caused variation in outcome and outcome-measure reporting.

### Interpretation

Randomised trials require a substantial investment of resources. Variation in outcomes and outcome measures limits the ability of trials to be combined with meta-analyses, which contributes to inevitable research waste, as identified in various areas of women’s health, including childbirth trauma, endometriosis and pre-eclampsia [91–94]. This systematic review is the first step in the development of a minimum data set, which will be known as a core outcome set. It will be developed with reference to methods described by the COMET initiative, Core Outcomes in Women’s and Newborn Health (CROWN) initiative and other core-outcome-set development studies, including those on endometriosis, pre-eclampsia, termination of pregnancy, Twin-Twin Transfusion Syndrome and neonatal medicine [95–99].

CHORUS is aiming to work towards a standardisation of outcomes and outcome measures and subsequently establish a minimum of standards in research and clinical practice. Chorus working groups are currently evaluating reported outcomes in all areas of urogyneacology and have been registered with the COMET (registration number 981, http://www.comet-initiative.org/studies/details/981) and CROWN initiatives. Each working group has carefully considered the scope of its work [100], and CHORUS will replicate the success of other international initiatives that have standardised outcome selection, collection and reporting across preterm birth research [101].

In the absence of a core outcome, we recommend QoL (incorporating sexual function), postoperative complications, patient and physician satisfaction and postoperative prolapse, bladder and bowel symptoms be collected across all anterior prolapse trials.

## Conclusion

Anterior-compartment prolapse trials report many different outcomes and outcome measures and often neglect to report important safety outcomes. Developing, disseminating and implementing a core outcome set will help address these issues.
